# Subtypes of physical frailty: Latent class analysis and associations with clinical characteristics and outcomes

**DOI:** 10.1038/srep46417

**Published:** 2017-04-11

**Authors:** Li-Kuo Liu, Chao-Yu Guo, Wei-Ju Lee, Liang-Yu Chen, An-Chun Hwang, Ming-Hsien Lin, Li-Ning Peng, Liang-Kung Chen, Kung-Yee Liang

**Affiliations:** 1 Aging and Health Research Center, National Yang Ming University, No. 155, Sec. 2, Linong St., Taipei City 11221, Taiwan; 2 Center for Geriatrics and Gerontology, Taipei Veterans General Hospital, No. 201, Sec. 2, Shi-Pai Rd., Taipei 11217, Taiwan; 3 Institute of Public Health, National Yang Ming University, No. 155, Sec. 2, Linong St., Taipei City 11221, Taiwan; 4 Department of Family Medicine, Taipei Veterans General Hospital, Yuanshan Branch, No. 386 Rongguang Rd., Yuanshan Township, Yilan County 264, Taiwan

## Abstract

Frailty is a well-recognized geriatric syndrome with various definitions and conceptual frameworks. This study aimed to use latent class analysis to discover potential subtypes of pre-frail and frail older people. Data from the I-Lan Longitudinal Aging Study (ILAS), a community-based cohort study was used for analysis. Latent class analysis was applied to characterize classes or subgroups with different frailty phenotypes among ILAS participants targeting older adults aged 65 and above, capable of completing a 6-meter walk, without severe major or life threatening diseases, and not institutionalized. Latent class analysis identified three distinct subgroups with different frailty phenotypes: non-mobility-type (weight loss and exhaustion), mobility-type frailty (slowness and weakness), and low physical activity. Comparing these groups with the robust group, people with mobility-type frailty had poorer body composition, worse bone health, poorer cognitive function, lower survival (hazard ratio: 6.82, p = 0.019), and poorer overall health outcomes (hazard ratio: 1.67, p = 0.040). People in the non-mobility-type group had poorer bone health and more metabolic serum abnormalities. In conclusion, mobility-type frailty was a better predictor of adverse outcomes. However, further investigation is needed to evaluate how these phenotypic subgroups may help in predicting prognosis or in developing interventions.

Frailty, a well-recognized geriatric syndrome that is characterized by loss of function and physiologic reserve, highlights the vulnerability older adults^1^,^2^. Several studies have shown that frailty may effectively predict adverse health outcomes and mortality^3^,^4^. A theoretical dynamic model that integrates biomedical and psychosocial perspectives has been proposed to evaluate frailty progression^5^. Though all frail elderly may share a final common pathway of functional decline due to dysregulated homeostasis of multiple organ systems^6^, the clinical presentations of frailty may differ greatly.

Previous studies have proposed various operational definitions and conceptual frameworks of frailty; however, despite different definitions in different study populations, the prevalence of frailty did not vary greatly^2^,^7^. Moreover, it is likely that no single operational definition of frailty will satisfy all experts, and previous studies failed to reach a consensus regarding the definition of frailty of clinical uses^8^. Due to the complex physiopathological process, multifaceted etiology, and diverse clinical phenotype of frailty, current studies categorize frailty into different functional domains, such as physical frailty, cognitive frailty, and social frailty^9^,^10^.

Although there are no universal criteria for assessing frailty, proper assessment of physical frailty and timely intervention may reduce subsequent disability, hospitalizations and mortality^11^,^12^. Instead of keeping creating new frailty criteria, we tried to focus on a more clearly defined and well-accepted term: “physical frailty”. Physical frailty has recently garnered extensive research interest, based on five phenotypic criteria proposed by Fried, *et al*. from the Cardiovascular Health Study (CHS)^13^: weakness; slowness; low level of physical activity; weight loss; and exhaustion. However, it was uncertain how each individual component and others were clustered. These five components may individually represent different underlying patho-etiology; however, their clustering may reveal common pathways underlying the frailty phenotype. Frailty component clusters may also suggest the existence of frailty subtypes that may help to clarify the trajectory for people with pre-frailty and frailty. Frailty subtypes may also improve the prediction of adverse outcomes and prognosis to devise effective intervention programs^14^. Therefore, this community-based cohort study in Taiwan employed latent class analysis (LCA), to identify distinct subgroups of subjects with different phenotype presentation(s) of pre-frail or frail status and investigated relationships between participant’s characteristics and health outcomes.

## Results

### Prevalence and clustering of frailty components

The prevalence of frailty and its individual components in ILAS were lower than in the CHS study population (Table 1), particularly exhaustion and slowness; however, differences in weight loss, low physical activity and weak grip strength between the ILAS and CHS cohorts were all within 5%. Table 2 summarizes the ILAS LCA results; to ascertain whether or not these components of frailty criteria aggregated into subgroups, we calculated the conditional probabilities of having each CHS frailty component within latent classes (Table 3). In two-class models, there was no evidence indicating that some components co-occurred preferentially in the specific class, whereas in three-class models, the prevalence of each component increased progressively across classes. In four-class models, excepting the expected class of clinically non-frail ILAS group as class 2, LCA showed class 1 to have very high conditional probability of low physical activity. Furthermore, class 3 had high probability of three CHS criteria including low physical activity, slowness and weakness; this class fit the CHS frailty definition best, though the prevalence differed from that obtained using CHS criteria (15.8% by LCA vs. 6.8% in ILAS). AIC favored the three-class model while BIC favored the two-class model. Results of the LCA four-class model further suggested that slowness and weakness may cluster as a unique phenotype subgroup, and that low physical activity may be another.

### Mobile, non-mobile and physically inactive frailty groups

**Figure 1:**
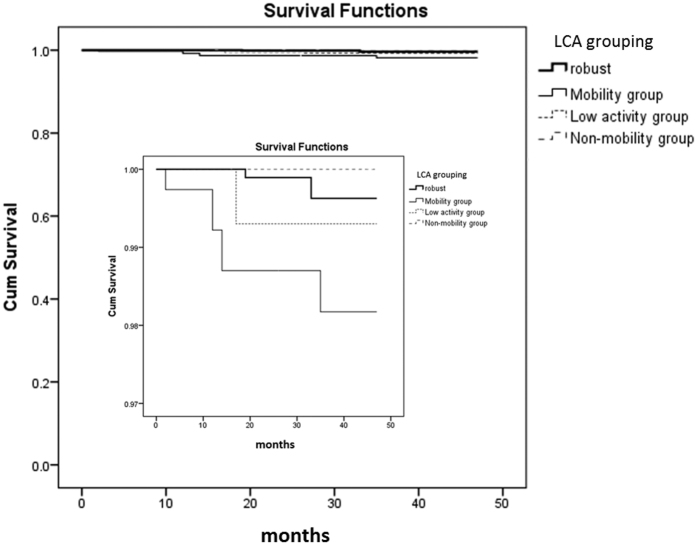
Kaplan-Meier survival curves for different subgroup in ILAS participants by LCA analysis.

Based on the results of the LCA three-class model, we categorized participants as either mobility-type or non-mobility type frailty, or low physical activity. Table 4 shows comparisons between different groups based on LCA. Compared to robust and other frailty subtypes, the mobility group was older, had greater waist circumference, lower appendicular skeletal muscle mass, the lowest hip T-score, poorer SMAF score functional status, and lower cognitive function based on MMSE adjusted for education level. Although the mobility group had statistically greater waist circumference, there were no significant differences in BMI or total body-fat percentage between groups. On the other hand, the non-mobility group had the most depressed mood, highest risk of malnutrition, and a lower hip T-score; they also had higher levels of HbA1c and LDL-C and lower HDL-C. Compared to the robust group, mobility type frailty had higher risk for mortality and poorer composite outcomes (Table 5); this effect was more significant when low physical activity was added to mobility type frailty compared with the robust group). However survival analysis of non-mobility frailty with or without low physical activity, and low physical activity only, showed no statistically difference compared to the robust group. Figure 1 showed the Kaplan-Meier survival curves for different subgroup in ILAS participants by LCA analysis.

### Adverse health outcomes

Only five participants were lost to follow-up due to changing address or being uncontactable by telephone. During average follow-up of 2.6 years, 117 outcome events were reported, including 10 emergency room visits, 57 hospitalizations, 28 falls, three long-term care facility placements, and 19 deaths.

## Discussion

To the best of our knowledge, this is the first evaluation of frailty phenotypes using LCA, which suggested that components of physical frailty aggregate as a syndrome. LCA showed that four probable subgroups captured heterogeneity in the frailty definition better than two or three; however, the prevalence of these subgroups were not consistent with clinically defined pre-frail and frail groups. Interestingly, the prevalence of class 1 was similar to the non-frail group in ILAS, but the prevalence of class 2 and class 3 were discordant with the pre-frail and frail groups. In the four-class LCA model, slowness and weakness frequently aggregated together; therefore, we proposed these two criteria as mobility-type frailty. On the other hand, weight loss and exhaustion did not show such strong aggregation; hence, we grouped these components as non-mobility type frailty. Consequently, we hypothesize that frailty can be categorized into mobility type and non-mobility type.

Xue *et al*. proposed a cycle of frailty based on the Women’s Health and Aging Study II^15^, in which any frailty component could initiate the frailty cycle, and different initiators may lead to different rates of frailty progression; the most common initiator was weakness. The study also suggested that weakness, slowness, and low physical activity often co-occur and precede exhaustion and weight loss^15^, which could partially explain our results.

### Mobility-type frailty

Lower muscle strength and/or physical performance may be attributed to loss of muscle mass or diminished muscle quality, which begin in middle-age. Various mechanisms suggested to explain these muscle changes including proteolysis, oxidative stress, dysregulation of inflammatory cytokines and hormones, physical inactivity, and undernutrition, all of which will further contribute to frailty through interactive pathways^16^,^17^. Manini *et al*. have attributed age-related decline in muscle strength to a combination of neurologic and muscular factors^18^.

Slowness (slow walking speed) and weakness (low grip strength) are frequently used to measure physical performance and muscle strength in older people^19^,^20^; however, slowness and weakness may also have a neurologic etiology. In our previous study, Huang *et al*. discovered that slowness and weakness with non-muscle etiology were strongly associated with cognitive impairment^21^. In addition, Wu *et al*. found that among all frailty components, slowness and weakness were the most significantly associated with cognitive impairment, and also that, rather than memory impairment, non-memory domains, such as executive dysfunction, appear early in the robust-prefrail-frail trajectory^22^. Accordingly, we propose that neurological degeneration may play a more important or an earlier role in physical decline than muscular degeneration itself, and that mobility-type frailty would further affect cognitive function, starting with non-memory domains. Congruently, another study reported that mobility dysfunction is often associated with cerebellar atrophy^23^.

In the ILAS cohort, worse mobility type frailty was associated with greater central obesity, lower appendicular skeletal muscle mass, the worst hip T-score, statistically higher comorbidity burden among all groups; this group also had poorer cognitive function, low physical performance, more people with hypertension and diabetes.

The association between aging and diminished walking speed and grip strength has been reported^24^,^25^, and both variables effectively predict poor health outcomes such as institutionalization and mortality^26^,^27^. Although there was a good correlation between the two physical measurements in frail individuals^28^, using either parameter alone showed a poorer prediction rate^29^,^30^.

### Non-mobility-type frailty

In this study, clustering between weight loss and exhaustion was not statistically significant; this may be because weight loss and exhaustion were quantified by self-reported questionnaire, and the prevalence was low in ILAS. Besides, a previous study has suggested that weight loss and exhaustion may occur later in the cycle or trajectory of frailty, long after the mobility type phenotype manifests^15^. In contrast to the muscle catabolic or neurological pathway leading to mobility type frailty, the underlying mechanism of non-mobility-type frailty may be even more complicated.

Weight loss, especially among people aged 70 years and older, is a risk indicator for current health problems such as underlying diseases, and mortality^31^. Therefore, underlying chronic diseases, chronic inflammation, and adverse circumstances including malnutrition, loss of fat-free mass, functional decline and impaired immunity should be carefully evaluated^32^,^33^. Accordingly, screening older adults with significant weight loss for frailty has been advocated^34^.

Exhaustion is considered to result from energy dysregulation^13^. In fact, women with weight loss and exhaustion as initial presentations are more likely to get worse^15^. In our analysis, participants with non-mobility frailty showed no remarkable difference in body composition, besides lower hip T-score, compared to the robust group. Interestingly, they had some metabolism-related declines, including poor nutrition status, higher HbA1c, higher LDL-C and lower HDL-C serum levels. They were also the most depressed group overall. Moreover, survival analysis of the non-mobility frailty group showed no statistical difference compared to the robust group.

### Low physical activity

Low physical activity may be the most ambiguous of the CHS frailty criteria. In this study (Table 3), low physical activity was isolated as one class in the four-class model. It can also combine with mobility-type phenotypes and result in a specific frailty group (class 3 in LCA four-class model). Women’s Health and Aging Study data also showed that in an LCA three-class model, concurrent weakness, slowness and low physical activity showed very high prevalence in a specific class, which they defined as frail^35^.

Though forming a specific LCA class, low physical activity actually broadly and strongly interacted with other frailty criteria. Slowness, weakness and skeletal muscle loss may curb older adults’ willingness to exercise, and decrease regular physical activity. Soon, diminished resting metabolism and total energy expenditure preempt undernourishment and further muscle mass loss and performance decline^1^,^5^,^31^. Physically inactivity also causes loss of muscle mass, by decreasing the rate of muscle synthesis or increasing muscle protein degradation^36^.

Furthermore, though criteria of the mobility-type subgroup and low physical activity may share similar underlying pathways, some studies have found that chronic multimorbidity and diseases strongly related to physical impairment can only explain a small part of reduced physical performance^37^.

Based on the Survey in Europe on Nutrition and the Elderly, Chin *et al*. found that unintentional weight loss was the most significant symptom associated with inactivity in frail individuals; physical inactivity alone or combined with weight loss strongly predicted less favorable health and nutritional characteristics and poorer physical functioning^38^. Other studies have also revealed the relationship between malnutrition, weight loss and inactivity among home-dwelling older people^39^. Low physical activity has been independently associated with activities of daily living and instrumental activities of daily living disability too ref.^40^.

Though many studies have shown that all five CHS frailty criteria have some correlation with poor clinical outcomes, we found mobility-type frailty to be more significantly associated with poor health outcomes and mortality, and the correlation was even stronger when mobility-type criteria were combined with low physical activity; however, non-mobility-type frailty did not show such a significant association with poor clinical outcomes.

## Conclusion

Given the laboratory findings in this study, wasting or chronic inflammatory processes may theoretically contribute to the non-mobility phenomenon. While non-mobility-type frailty has a complicated underlying mechanism, mobility-type frailty was probably largely due to neurodegeneration; such physical frailty manifested earlier in the frailty trajectory, and was more associated with cerebellar degeneration.

The underlying mechanisms of frailty are complex, and its trajectories of clinical progression vary widely, even when we only consider physical frailty. We hypothesize that frailty can be subdivided into new phenotypic categories of mobility-type and non-mobility-type frailty. However, further investigation is needed to ascertain the additional value of these subgroups for predicting adverse outcomes or helping to develop efficient interventions.

## Methods

### Study subjects and design

The I-Lan Longitudinal Aging Study (ILAS) is a research cohort of community-dwelling residents aged 50 years or more from I-Lan (Yilan) County, in Northeast Taiwan. Residents were randomly sampled through the household registrations of the county government. Selected residents were invited to participate by mail or telephone invitations from the research team, and were enrolled when they had fully consented and agreed for participation. The inclusion criteria were: (i) inhabitants who then lived in I-Lan County without a plan to move in the near future; and (ii) inhabitants aged 50 years or older. Any respondents that met any one of the following conditions were excluded from the study: (i) the respondent was unable to communicate with the interviewer and grant an interview; (ii) the respondent had a poor function status, which could lead to a fail in evaluation, such as unable to complete a 6-meter timed walk within a reasonable period of time; (iii) the respondent had a limited life expectancy (in general, <6 months) because of major illnesses; (iv) currently institutionalized people. The design and participant selection have been described previously^41^. This substudy investigated the complex interrelationships between aging, frailty, sarcopenia, and cognitive decline; the specific aim was to identify potential subgroups of the frailty phenotype and to compare demographic characteristics and laboratory test results between subgroups. A written informed consent was obtained from every participant. The Institutional Review Board of National Yang Ming University approved the ILAS study protocol. The design and procedures of the study were carried out in accordance with the principles of the Declaration of Helsinki.

### Demography, physical examinations and laboratory measurements

Study participants completed a questionnaire to elicit information on their demographic characteristics, socioeconomic status, anthropometric measurements, medical history, functional performance, and burden of chronic diseases. Comprehensive functional assessment included the Center for Epidemiologic Studies Depression Scale (CES-D) for mood status^42^, Mini-Mental State Examination (MMSE) for cognitive function^43^, Functional Autonomy Measurement System (SMAF) including activities of daily living^44^, and Mini-nutrition Assessment (MNA)-short form for nutritional status^45^. The burden of chronic diseases was evaluated using Charlson Comorbidity Index.

All participants provided an overnight, 10-hour, fasting blood sample; serum concentrations of albumin, creatinine, low-density and high-density lipoprotein cholesterol (LDL-C & HDL-C), were analyzed automatically (ADVIA 1800, Siemens, Malvern, PA, USA). Other cardiometabolic-related measurements included whole-blood glycated hemoglobin (HbA1c) and high-sensitivity C-reactive protein (hs-CRP). Hormone profiling included growth hormone, dehydroepiandrosterone sulfate (DHEA-S), insulin-like growth factor-1 (IGF-1) and 25-hydroxyvitamin D (25(OH)D).

### Frailty phenotype

Modified Fried’s criteria were used to define physical frailty, which comprised exhaustion, weakness, slowness, physical inactivity, and weight loss^13^; the published criteria were modified by using the baseline measurements of ILAS participants – Table 1 shows the cross-validated data. Exhaustion was defined using two items of the CES-D questionnaire. Weakness was defined by low handgrip strength; slowness was defined by slow walking speed; and physical inactivity was gaged using the International Physical Activity Questionnaire^46^; subjects whose performance level was lower than the gender-specific lowest quintile of the study population were designated weak and/or slow and/or physically inactive according to the initial definition in the CHS study. Weight loss was defined as having either unintended weight loss exceeding 5% of body weight in the past year, or 3 kg within 3 months. Individuals fulfilling three or more CHS criteria were classified as frail, and those meeting one or two criteria were assigned pre-frail status; those negative for all five criteria were considered robust.

### Body composition and bone density

All participants received a whole-body dual-energy X-ray absorptiometry (DXA) scan (Lunar Prodigy instrument, GE Healthcare, Madison, WI, USA). Total body-fat mass percentage and appendicular skeletal muscle mass (ASM), defined as the summed muscle mass of four limbs, were recorded; bone mineral density at the bilateral hip joints was measured, and the T-score was calculated.

### Adverse health outcomes

Three-monthly follow-up telephone interviews were conducted to record adverse health outcomes after enrollment; these included falls, unexpected emergency department visits, hospitalizations, institutionalizations, and mortality.

### Missing Data

Participants with a missing value on any of the variables were excluded from the analysis. There was no statistically difference on age, gender, and other key variables between the studied and excluded population.

### Statistical analysis

LCA was used to identify distinct participant subgroups with different frailty phenotypes; LCA is a subset of structural equation modeling, which is used to detect homogeneous subgroups within a larger heterogeneous population^47^,^48^: the subtypes, termed “latent classes”, may present according the disease entities or patterns of association in the respective phenotypes. To measure co-occurrence, we tabulated frailty criteria to assess the convergent validity; LCA was then used to determine the number of classes or subgroups^49^. In addition, following reported recommendations, indices-of-fit of the model with different numbers of classes were compared using Pearson’s chi square^50^, the Akaike Information Criterion (AIC)^51^, and the Bayesian Information Criterion (BIC)^52^.

Improvements-of-fit of the models were evaluated from two up to four classes. Based on LCA results, slow and/or weak participants were statistically significantly clustered and were therefore categorized as the “mobility group”. Weight loss and/or exhaustion were also more likely to be clustered, and were categorized as the “non-mobility group”. The differences between groups of clinical characteristics and laboratory measurements were compared by one-way ANOVA with post-hoc analysis for continuous variables; comparisons between categorical data were made by χ^2^-test when appropriate. The Cox proportional hazard model was used to compare mortality and poor health outcomes between the mobility and non-mobility groups. Non-mobility and mobility group subjects with and without low physical activity and participants with low physical activity only, were compared to the robust group.

Statistical and LCA analysis, description of characteristics, testing differences and survival analyses were performed using SPSS 18.0 software (SPSS Inc., Chicago, IL). A two-tailed p value of ≤0.05 was considered statistically significant.

## Additional Information

**How to cite this article:** Liu, L.-K. *et al*. Subtypes of physical frailty: Latent class analysis and associations with clinical characteristics and outcomes. *Sci. Rep.* **7**, 46417; doi: 10.1038/srep46417 (2017).

**Publisher's note:** Springer Nature remains neutral with regard to jurisdictional claims in published maps and institutional affiliations.

## Figures and Tables

**Table 1:** Frailty-defining criteria in ILAS and CHS.

Characteristics	ILAS	%	CHS				
	Definition		Definition				%
Weight loss	Lost >5 kg (5%) unintentionally in last year, or Lost >3 kg unintentionally within 3 months	5.3	Lost >10 lbs unintentionally in last year				7.3
Exhaustion	As for CHS	4.0	Self-report of either of: (i) felt that everything I did was an effort in the last week, or (ii) could not get going in the last week				21.3
Low physical activity	The lowest gender-specific 20% of study population (IPAQ score)	20.0	A version of the Minnesota Leisure Time Activities Questionnaire				24.1
			Women < 270		Men < 383		
Slowness	Walking 6 meters: The lowest gender-specific 20% of study population	23.6	Time to walk 15 feet (4.57 m)				38.0
			Women		Men		
			≤159 cm high	>159 cm high	≤173 cm high	>173 cm high	
			≥7 s	≥6 s	≥7 s	≥6 s	
Weakness	Grip strength: The lowest gender-specific 20% of study population	21.6	Grip strength (kg)				26.2
			Women		Men		
			BMI ≤ 23	≤17 kg	BMI ≤ 24	≤29 kg	
			BMI 23.1–26	≤17.3 kg	BMI 24.1–28	≤30 kg	
			BMI 26.1–29	≤18 kg			
			BMI > 29	≤21 kg	BMI > 28	≤32 kg	
**Overall frailty status**	Robust	52.7	Robust				33.2
	Pre-frail	40.5	Pre-frail				55.2
	Frail	6.8	Frail				11.6

ILAS, The I-Lan Longitudinal Aging Study; CHS, Cardiovascular Health Study; BMI, body mass index (kg/m^2^); IPAQ, International Physical Activity Questionnaire.

**Table 2:** Frailty Criteria Patterns and Latent Class Analysis Fit: ILAS.

Parameter estimates	Measurement items (in order): Weight loss/Exhaustion/Low activity/slowness/weakness	Observed count	Pattern frequncies expected		
	Criterion: No (N) or Yes (Y)		2-Class Model	3-Class Model	4-Class Model
6 Most frequently observed nonfrail patterns	NNNNN	970	1023.7	1097.8	1066.4
	NNYNN	166	170.2	185.7	193.0
	NNNYN	144	141.7	120.8	95.3
	NNNNY	141	143.4	135.1	121.3
	NNNYY	100	76.3	81.6	86.7
	NNYYN	47	46.1	42.1	39.8
6 Most frequently observed frail patterns	NNYYY	73	52.2	58.9	70.1
	NYYYY	10	7.7	10.2	10.7
	NYNYY	9	9.9	8.5	8.5
	NYYYN	9	4.8	7.3	9.6
	YNNYY	7	6.2	7.0	4.2
	NYYNY	4	2.3	3.6	3.5
**Latent class model fit statistics**					
Pearson chi square			27.7 (p < 0.0001)	12.2 (p = 0.007)	5.1 (p = 0.274)
AIC			6840	6837	6843
BIC			6901	6930	6970

ILAS, The I-Lan Longitudinal Aging Study; AIC, Akaike Information Criterion; BIC, Bayesian Information Criterion.

**Table 3:** Conditional Probabilities of Meeting Criteria Within Latent Frailty Classes: ILAS*.

Rho estimate	3-class model			4-class model			
	Class 1 (Non-frail)	Class 2	Class 3	Class 1	Class 2 (Non-frail)	Class 3	Class 4
Class prevalence (%)	55.5	25.0	19.5	3.6	55.4	15.8	25.3
Weight loss	0.0257	0.1072	0.0600	0.1030	0.0148	0.0532	0.1284
Exhaustion	0.0190	0.0003	0.1494	0.2007	0.0201	0.1350	0.0004
Low physical activity	0.1633	0.0152	0.5402	0.8851	0.1592	0.4657	0.0250
Slowness	0.0048	0.3499	0.7497	0.2427	0.0078	0.9442	0.2930
Weakness	0.0593	0.2749	0.5898	0.4362	0.0930	0.6130	0.2081

ILAS, The I-Lan Longitudinal Aging Study. *Per class and criterion: Estimated proportion in class exhibiting the criterion.

**Table 4:** Comparisons between different groups based on LCA.

Variable	Robust	Non-mobility group*	Mobility group^†^	Low activity	p-value
Number	970	61	385	166	
Age (years)	60.7 ± 7.5	61.5 ± 7.2	69.0 ± 8.9	60.9 ± 7.9	<0.001
Sex (male %)	45.8	45.9	52.2	46.4	0.193
Smoking (%)	16.6	23.0	19.0	18.7	0.095
WC (cm)	83.8 ± 9.4	85.8 ± 9.5	85.9 ± 10.1	84.5 ± 9.9	0.004
BMI (kg/m^2^)	24.8 ± 3.5	25.2 ± 3.8	24.8 ± 3.7	25.1 ± 3.5	0.569
Total body fat (%)	31.5 ± 8.7	31.6 ± 9.6	31.0 ± 8.8	32.9 ± 7.2	0.133
ASM (kg)	18.3 ± 4.3	18.6 ± 4.4	17.3 ± 3.7	18.1 ± 4.1	<0.001
Hip T-score	−0.66 ± 1.04	−0.86 ± 1.14	−1.15 ± 1.09	−0.67 ± 1.03	<0.001
SMAF	−0.01 ± 0.15	0.0 ± 0.0	−0.11 ± −0.68	−0.01 ± 0.08	<0.001
MMSE	26.8 ± 3.0	26.5 ± 3.0	24.2 ± 4.0	26.8 ± 3.4	<0.001
CES-D	1.5 ± 2.6	4.5 ± 6.0	2.3 ± 3.6	1.7 ± 3.0	<0.001
MNA-SF	13.4 ± 0.8	12.9 ± 1.6	13.4 ± 0.9	13.6 ± 0.8	<0.001
CCI	0.7 ± 1.1	0.9 ± 1.2	1.4 ± 1.4	0.7 ± 1.0	<0.001
Hypertension (%)	34.64	37.70	50.13	39.76	<0.001
Diabetes (%)	13.40	18.03	19.74	15.66	0.031
Dyslipidemia (%)	6.19	16.39	11.17	6.02	0.001
**Metabolic parameters**					
Albumin (mg/dl)	4.6 ± 0.2	4.5 ± 0.3	4.4 ± 0.3	4.6 ± 0.2	<0.001
HbA1c (%)	6.0 ± 0.9	6.2 ± 1.5	6.1 ± 1.0	6.1 ± 1.2	0.012
LDL-cholesterol (mg/dl)	120.3 ± 32.6	132.5 ± 33.4	114.8 ± 31.0	118.3 ± 33.8	<0.001
HDL-cholesterol (mg/dl)	55.8 ± 13.9	53.1 ± 14.5	53.6 ± 13.1	54.0 ± 13.6	0.030
Creatinine (mg/dl)	0.8 ± 0.3	0.8 ± 0.4	0.8 ± 0.2	0.9 ± 0.5	0.371
hs-CRP (mg/dl)	0.2 ± 0.4	0.2 ± 0.3	0.2 ± 0.5	0.2 ± 0.4	0.221
**Hormones and Endocrines**					
Growth hormone (ng/ml)	0.8 ± 1.4	0.9 ± 1.4	0.6 ± 1.1	0.8 ± 1.2	0.131
Free Androgen Index (%)	17.4 ± 18.9	19.8 ± 22.6	17.2 ± 16.7	17.9 ± 19.0	0.780
DHEA-S (μg/dl)	113.4 ± 71.1	101.2 ± 57.3	91.9 ± 60.2	119.9 ± 74.7	<0.001
IGF-1 (ng/ml)	144.3 ± 57.7	145.6 ± 51.5	119.8 ± 49.7	144.5 ± 60.4	<0.001
25(OH)D (ng/ml)	23.1 ± 6.5	23.3 ± 6.0	25.2 ± 8.5	21.3 ± 6.1	<0.001

*Non-mobility group: participants with weight loss, exhaustion or both. ^†^Mobility group: participants with weakness, slowness or both. WC, waist circumference; BMI, body mass index; ASM, appendicular skeletal muscle mass; SMAF, the Functional Autonomy Measurement System; MMSE, Mini-Mental State Examination; CES-D, the Center for Epidemiologic Studies Depression Scale; MNA-SF, Mini-nutrition assessment-short form; CCI, Charlson Comorbidity Index; HbA1c, glycated hemoglobin; LDL, low-density lipoprotein; HDL, high-density lipoprotein; hs-CRP, high-sensitive C-reactive protein; DHEA-S, dehydroepiandrosterone sulfate; IGF-1, insulin-like growth factor-1; 25(OH)D, 25-hydroxyvitamin D.

**Table 5:** Cox proportional hazard ratios for times until death and until other poor health outcomes: ILAS.

Frail state	Poor health Outcomes				
	Number/death n	Death		Total	
		HR (95% CI)	p-value	HR (95% CI)	p-value
Robust	970/2	1.00 (reference)		1.00 (reference)	
Non-mobility group*	61/0	N/A	0.988	1.00 (0.31–3.25)	0.999
Mobility group^†^	385/6	6.82 (1.37–33.85)	0.019	1.67 (1.02–2.74)	0.040
Low activity	166/1	3.76 (0.34–41.60)	0.281	0.71 (0.25–1.99)	0.514
Non-mobility + Low activity	14/0	N/A	0.994	1.55 (0.21–11.27)	0.667
Mobility + Low activity	154/5	17.16 (3.33–88.51)	0.001	2.67 (1.47–4.84)	0.001

*Non-mobility group: participants with weight loss, exhaustion or both. ^†^Mobility group: participants with weakness, slowness or both. HR, hazard ratio; CI, confidence interval, ILAS, The I-Lan Longitudinal Aging Study; N/A, not available.
